# The *Arabidopsis* Gene *zinc finger protein 3(ZFP3)* Is Involved in Salt Stress and Osmotic Stress Response

**DOI:** 10.1371/journal.pone.0168367

**Published:** 2016-12-15

**Authors:** Aidong Zhang, Dongdong Liu, Changmei Hua, An Yan, Bohan Liu, Minjie Wu, Yihua Liu, Linli Huang, Imran Ali, Yinbo Gan

**Affiliations:** Zhejiang Key Lab of Crop Germplasm, Department of Agronomy, College of Agriculture and Biotechnology, Zhejiang University, Hangzhou, China; National Taiwan University, TAIWAN

## Abstract

Plants are continuously challenged by various abiotic and biotic stresses. To tide over these adversities, plants evolved intricate regulatory networks to adapt these unfavorable environments. So far, many researchers have clarified the molecular and genetic pathways involved in regulation of stress responses. However, the mechanism through which these regulatory networks operate is largely unknown. In this study, we cloned a C_2_H_2_-type zinc finger protein gene *ZFP3* from *Arabidopsis thaliana* and investigated its function in salt and osmotic stress response. Our results showed that the expression level of *ZFP3* was highly suppressed by NaCl, mannitol and sucrose. Constitutive expression of *ZFP3* enhanced tolerance of plants to salt and osmotic stress while the *zfp3* mutant plants displays reduced tolerance in *Arabidopsis*. Gain- and Loss-of-function studies of *ZFP3* showed that *ZFP3* significantly changes proline accumulation and chlorophyll content. Furthermore, over-expression of *ZFP3* induced the expressions of stress-related gene *KIN1*, *RD22*, *RD29B* and *AtP5CS1*. These results suggest that *ZFP3* is involved in salt and osmotic stress response.

## Introduction

Plants are constantly challenged by a variety of stresses such as high salinity, extreme temperature and drought [1,2]. Environmental stress has been one of the major factors affecting crops yield. These stresses cause huge losses in some cases is as high as 50% of the total yield of some major crops [1,3,4]. To cope with these stressful conditions, plants have evolved an intricate regulatory mechanism [5], which is mostly regulated through activation or inhibition of the expression of a series of transcription factors [5–7]. In the past few decades, a number of transcription factors have been found to participate in physiological responses of plants to abiotic stresses. These different transcription factors involved in abiotic stress generally belong to a large family which include C-repeat binding factors (CBFs), dehydration responsive element binding factors (DREBs), ethylene-responsive element binding factor (ERF), AP2/EREB, basic domain-leucine zipper (bZIP), NAC, MYB, MYC, WRKY and zinc finger proteins [6–12].

High salt stress can cause osmotic and ionic stress [1,13]. Salt stress involved in ionic homeostasis pathway was discovered by cloning the salt overly sensitive (*SOS*) genes. Mutations in *SOS* genes make plants more sensitive to Na^+^ stress [14]. SOS3, a calcineurin B-like protein, can bind to second messenger calcium and interact with SOS2, which is a serine / threonine protein kinase. Then, SOS3-SOS2 complex will activate SOS1, which is a plasma membrane Na^+^ / H^+^ antiporter [14,15]. High salt-induced osmotic stress pathway has not been fully explored especially for the ABA-independent pathway [15]. It was reported that several protein kinases, which are similar to MAPK components and a SLN1-like histidine protein kinase were also involved in osmotic stress sensing or signaling [14].

Zinc Finger Proteins (ZFPs) play significant roles in physiological and biochemical processes of plants, such as hormone signal transduction, DNA/RNA combination, morphogenesis, transcription regulation, trichome and root hair development, biotic and abiotic responses [16–23]. Several studies have reported the involvement of ZFPs in regulation of abiotic stress responses in plant. For instance, overexpression of *Zat10* or knockout and RNAi plants of *Zat10* can both increase the tolerance of osmotic and salinity stresses which suggest that it plays an important role as a negative and positive regulator [24]. Overexpression of Tamarix hispida zinc finger protein *ThZFP1* in *Arabidopsis* showed more tolerance to salt and osmotic stress by increasing POD and SOD activities, proline content and their relative genes such as *delta-pyrroline-5-carboxylate synthetase* (*P5CS*), *superoxide dismutase* (*SOD*) and *peroxidase* (*POD*) [17]. In *Arabidopsis*, four ZPT2-related C2H2-type zinc finger proteins (AZF1, AZF2, AZF3 and STZ) are all involved in salinity and abscisic acid stress by interaction with the EP2 sequence [25]. A zinc finger transcription factor ART1 increased tolerance to Aluminum via affecting the expression level of Al tolerance related genes in rice [26]. Overexpression of *O*.*sativa subspecies indica* stress-associated gene (*OSISAP1*) in tobacco revealed strong tolerance to cold, dehydration and salt stress [2]. Zat7, a Cys2/His2-type zinc finger protein in *Arabidopsis*, also play a role in salt tolerance by its EAR-motif. A deletion or a mutation of the EAR-motif abolishes this gene’s function [27].

On the basis of the number and order of cysteine residues and histidine residues in finger secondary structure, ZFPs are divided into nine types, including C2H2, C8, C6, C3HC4, C2HC, C2HC5, C4, C4HC3 and CCCH type [17]. Cys2/His2-type zinc finger, also known as classical or TFIIIA-type finger was mainly found in eukaryotes. Their key features are CX_2-4_CX_12_HX_3-5_H (C represents cysteine, H represents histidine, X represents any amino acid), which includes about 30 amino acids consisting of two antiparallel β hairpin and one α helix. In this structure, two pairs of conserved cysteine residues and histidine residues combine with a zinc ion tetrahedrally [28]. It is reported that each TFIIIA-type finger has one to four zinc fingers. Two adjacent zinc fingers are separated by different length of long spacer in plant. Nevertheless, Cys2/His2-type fingers are mostly clustered and separated by short length of spacers (six to eight amino acids), which are called HC link in yeast and animals [29]. ZFPs exert their function by integrating with cis-acting element in promoter region of specific target genes [9,30]. The first C2H2-type zinc finger gene, *ZPT2-1* (*EPF1*), was found in petunia. It can integrate with the promoter of *EPSPS* [30]. Since then, at least 40 TFIIIA zinc finger genes were cloned from a variety of plants, including petunia, Arabidopsis, soybean, pepper and rice [9,18–23,29].

C2H2 transcription factor, *ZFP3* (*AT5G25160*), was previously shown to regulate light and ABA signaling in seeds germination and plant development in *Arabidopsis* [31]. In this study, we investigate whether it plays a role in abiotic stress. Constitutive expression of *ZFP3* enhances tolerance of plants to salt and osmotic stress while the *zfp3* mutant plants displays reduced tolerance in *Arabidopsis*. Our results further demonstrated that overexpression of *ZFP3* could increase proline accumulation, chlorophyll content and the gene expressions of stress-related genes (*KIN1*, *RD22*, *RD29B* and *AtP5CS1*) in regulating salt and osmotic stress tolerance in *Arabidopsis*.

## Materials and Methods

### Plant materials and growth conditions

*Arabidopsis thaliana* ecotype Columbia Col-0 was used as the control in all experiments. The plants were grown in an environmentally controlled growth chamber programmed for cycles of 16 hours of light (95 μmol m^-2^ second^-1^, 22°C), 8 hours dark (18°C) and relative humidity of 70–80% as we previously described [20,21]. *Arabidopsis* seeds were surface-sterilized with 5% NaClO: water (v/v) for 8 min and washed five times with distilled water. Seeds were plated on Murashige and Skoog (MS) medium and vernalize at 4°C for 3 days in dark before being transferred to the growth chamber with growth condition described previously [23].

### Isolation of a *zfp3* loss-of—function mutant

A *zfp3* mutant was identified from the Nottingham *Arabidopsis* Stock Centre (NASC; catalogue number N379591). Homozygous mutant was confirmed by screening on MS medium containing 5.25 mg/L sulfadiazine.

### Construct cloning

For overexpression construct, sequences were first inserted into the pENTR-1A vector (Invitrogen) and then recombined into the destination vector using the Gateway LR reaction (Invitrogen) [20]. The destination vector was obtained from VIB (Flanders Interuniversity Institute for Biotechnology) as described before [18,20,21]. For cloning the overexpression construct, the gene-specific fragment was amplified from cDNA with primers containing *SalI* and *NotI* restriction sites. Then, the fragment was purified with a gel extraction kit (Axygen) before restriction digestion and cloning. The primers, which used for *ZFP3* overexpression, were as follows: FP (5'-GCGCGTCGACAATCATCTCCTGAAAAAATCTATC-3'), RP (5'-TGGCGGCCGCACTAACAATCACATGAAAAAACAG-3'). The construct in binary vector was transferred into *Agrobacterium tumefaciens* Stain GV3101, which was then used to transform *Arabidopsis* thaliana ecotype Columbia Col-0 by the floral-dip method as described by Clough and Bent (1998) [32]. Transgenic seeds were first selected on Murashige and Skoog medium containing Kanamycin (50mg/L) and then confirmed by PCR with corresponding primers.

### Stress treatment

For phenotypical analyses, seeds were sown on the 1/2 MS medium containing different concentrations of NaCl, mannitol and sucrose. Emergence of radicles from the seed coat was used as the standard of seed germination. The germination rate was calculated as the percentage of number of germinated seeds/number of total seeds, which was recorded daily for seven days.

For the root length measurement, seeds were cultured on 1/2 MS medium and grown on vertically placed plates for 3 days under normal condition and uniform seedlings were then transferred to 1/2 MS mediums with or without different concentrations of NaCl, mannitol and sucrose. After 7 days treatment, photos were taken and the root lengths of different plants were measured by Image-Pro plus 6.0.

For gene expression analysis, 10 days seedlings were transferred onto filter paper saturated with 0, 200mM NaCl, 250mM sucrose and 300mM mannitol in MS liquid media. The samples were harvested at different time points.

### RNA extraction and real-time PCR

Total RNA was extracted from *Arabidopsis* plants using Trizol (Takara). First-strand cDNA was synthesized from 2 μg of total RNA by M-MLV transcriptase (Promega) and oligo (dT18) primer according to the manufacturer’s instructions [21,23]. For real-time PCR analysis, reaction was performed on 2μl 4 times diluted cDNA templates, 10μl SYBR Green PCR mix (Takara) and 0.4μl of each primers (200 nM final concentration) in 20μl reactions. qRT-PCR was initiated with denaturation at 95°C for 30 s, followed by 40 cycles each consisting of denaturation at 95°C for 5 s and annealing/extension at 60°C for 30 s. To determine the specificity of the reaction, a melting curve analysis of the product was performed immediately after the final PCR cycle by increasing the temperature from 60 to 90°C at 0.2 to 1 s. *UBQ10* (*At4g05320*) and *TUB2* (*At5g62690*) were used as the endogenous control gene for qRT-PCR analyses. Relative expression of target genes was calculated as previously described [23,33]. The primers used are listed in Table 1.

**Table 1:** Primers used for quantitative real-time PCR, Arabidopsis thaliana ecotype Columbia (Col-0) was used for this study.

Gene	Sequence	Reference
*ZFP3*	LP:5’-GTCAGCCTCAGCTTTTGGAC-3’	[31]
	RP:5’-AGTGACCACCAAACCCGTTA-3’	[31]
*UBQ10*	LP:5’-GGTTCGTACCTTTGTCCAAGCA-3’	[23]
	RP:5’-CCTTCGTTAAACCAAGCTCAGTATC-3’	[23]
*KIN1*	LP:5’-AAGAATGCCTTCCAAGCCGGTCAG-3’	[15]
	RP:5’-TACACTCTTTCCCGCCTGTTGTGC-3’	[15]
*RD22*	LP:5’-ATAATCTTTTGACTTTCGATTTTACCG-3	[15]
	RP:5’-CTTGGACGTTGGTACTTTTCTCG-3	[15]
*RD29B*	LP:5’-AGAAGGAATGGTGGGGAAAG-3’	[15]
	RP:5’-CAACTCACTTCCACCGGAAT-3’	[15]
*AtP5CS1*	LP:5’-TAGCACCCGAAGAGCCCCAT-3’	[15]
	RP:5’-TTTCAGTTCCAACGCCAGTAGA-3’	[15]
*TUB2*	LP:5’-ATCCGTGAAGAGTACCCAGAT-3’	[34]
	RP:5’-AAGAACCATGCACTCATCAGC-3’	[34]

### Determination of chlorophyll content

The chlorophyll was extracted in 95% ethyl alcohol from the fresh leaves at darkness. Absorbance of solution was measured at wavelength of 470, 649 and 665 nm with spectrophotometer (UV-2450, Shimadzu, Japan) to analysis the content of chlorophyll(a) and chlorophyll(b), respectively.

### Determination of proline content

Proline content of 10 days *Arabidopsis* seedlings was determined according to the procedure of Bates *et al*. (1973) [35].

### Statistical analyses

All data displayed in figures were analyzed by means of ANOVA for significance using SPSS, version 14.0 software (SPSS Inc., Chicago, USA). Student’s t-test was calculated to evaluate the difference at the probability of either 5% (*, P < 0.05 with significant level) or 1% (**, P < 0.01 with significant level) [33].

## Results

### *ZFP3* affects the response to salt and osmotic stress

**Fig 1:**
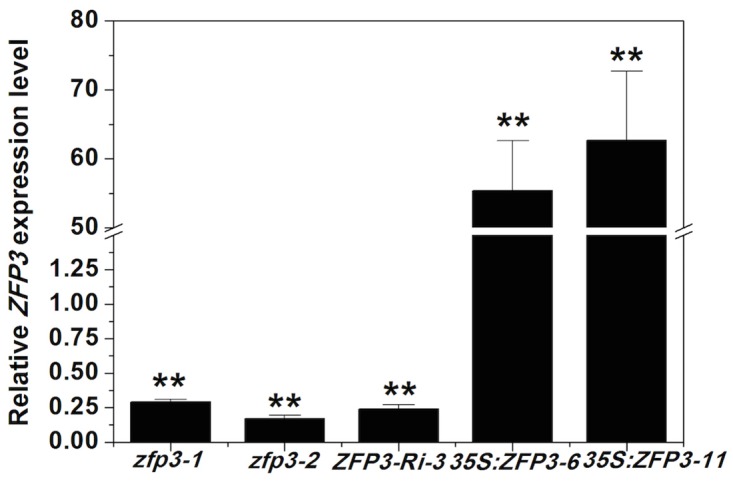
Relative expression levels of *ZFP3* in *zfp3* and *35*:*ZFP3* line. Transcript abundance was measured by real-time RT-PCR and the wild type or the corresponding control values were set at 1. Error bars indicate SD. **, *P*<0.01(Student’s t-test).

**Fig 2:**
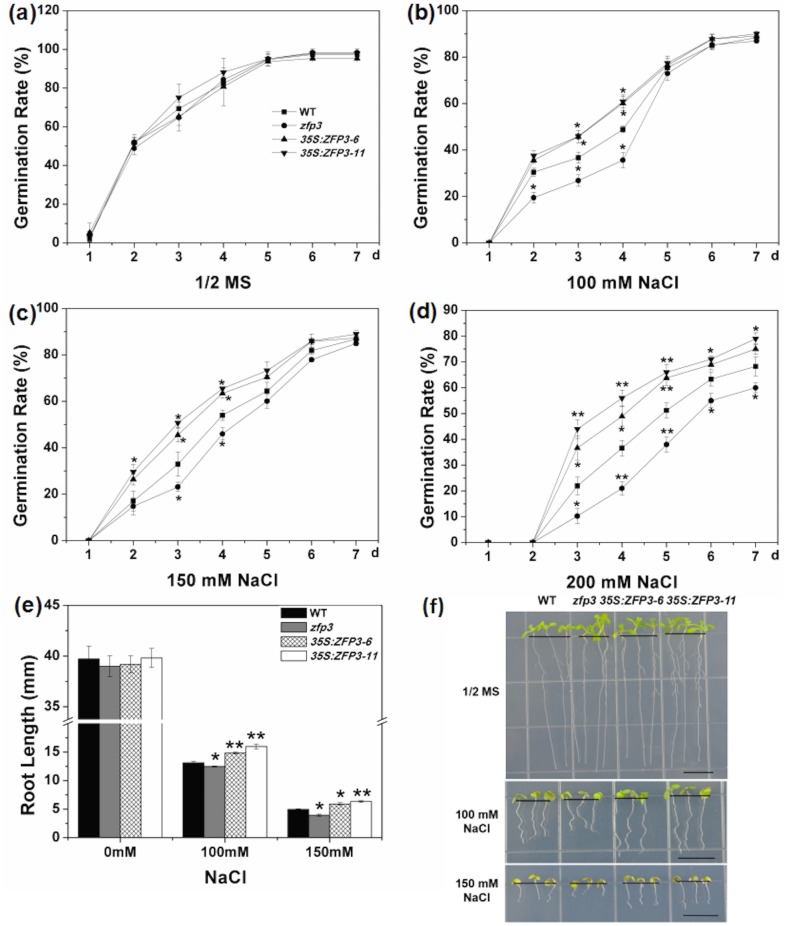
Phenotypes of *zfp3* mutant and *35S*:*ZFP3* treated with different concentrations of NaCl. **(a to d)** Seed germination rate of WT, *zfp3* and *35S*:*ZFP3* on 1/2 MS medium containing different concentrations of NaCl. Each curve represents an average of three replicates. **(e)** Effect of different NaCl treatment on root length of WT, *zfp3* and *35S*:*ZFP3*. **(f)** Phenotype of WT, *zfp3* and *35S*:*ZFP3* seedlings under NaCl treatment. Each experiment as repeated at least three times with identical results. Bars = 10 mm. Error bars indicate SD. *, *P*<0.05; and **, *P*<0.01(Student’s t-test).

**Fig 3:**
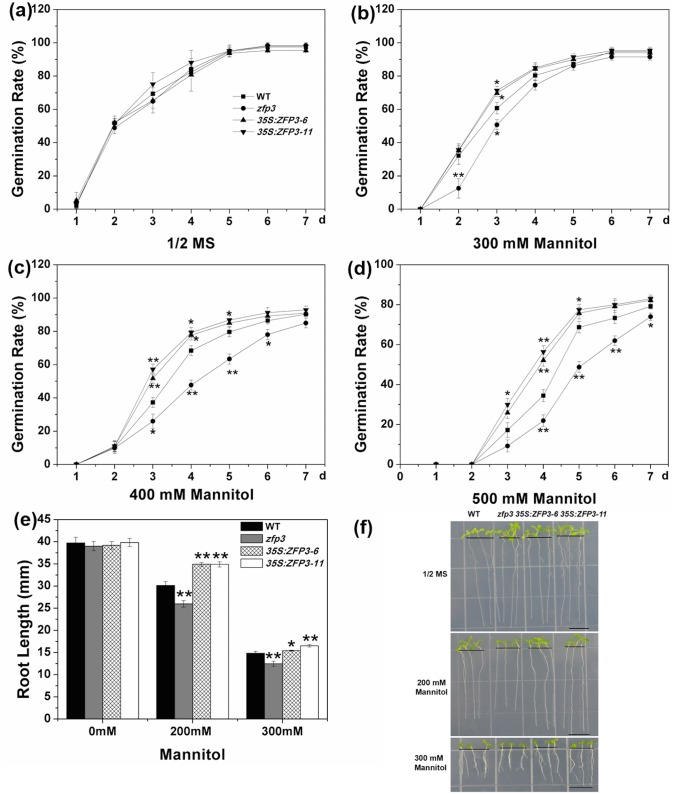
Phenotypes of *zfp3* mutant and *35S*:*ZFP3* treated with different concentrations of mannitol. **(a to d)** Seed germination rate of WT, *zfp3* and *35S*:*ZFP3* on 1/2 MS medium containing different concentrations of mannitol. Each curve represents an average of three replicates. **(e)** Effect of different mannitol treatment on root length of WT, *zfp3* and *35S*:*ZFP3*. **(f)** Phenotype of WT, *zfp3* and *35S*:*ZFP3* seedlings under mannitol treatment. Each experiment as repeated at least three times with identical results. Bars = 10 mm. Error bars indicate SD. *, *P*<0.05; and **, *P*<0.01(Student’s t-test).

**Fig 4:**
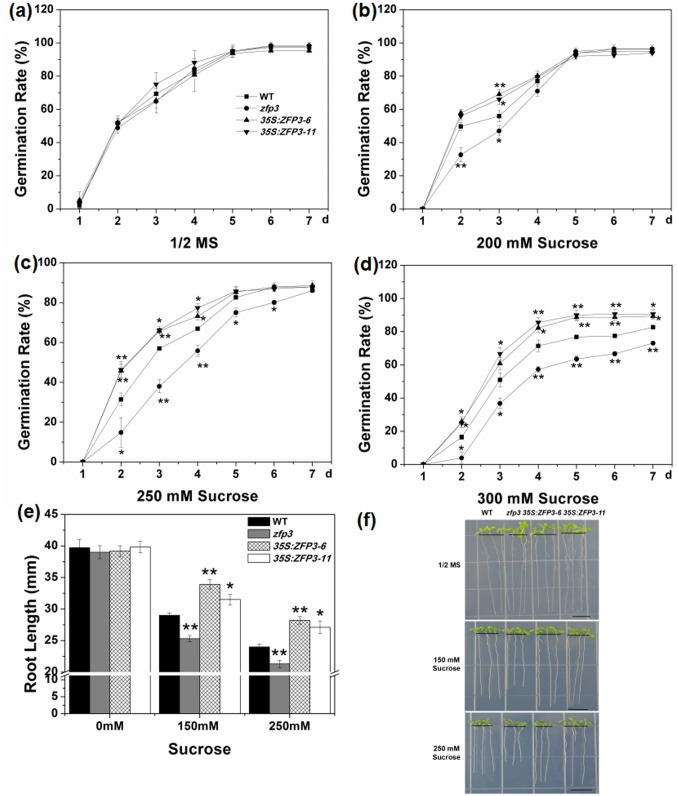
Phenotypes of *zfp3* mutant and *35S*:*ZFP3* treated with different concentrations of sucrose. **(a to d)** Seed germination rate of WT, *zfp3* and *35S*:*ZFP3* on 1/2 MS medium containing different concentrations of sucrose. Each curve represents an average of three replicates. **(e)** Effect of different sucrose treatment on root length of WT, *zfp3* and *35S*:*ZFP3*. **(f)** Phenotype of WT, *zfp3* and *35S*:*ZFP3* seedlings under sucrose treatment. Each experiment as repeated at least three times with identical results. Bars = 10 mm. Error bars indicate SD. *, *P*<0.05; and **, *P*<0.01(Student’s t-test).

To determine the effects of *ZFP3* in abiotic stress responses, a *zfp3* mutant (catalog no. N379591) was ordered and screened from the Nottingham *Arabidopsis* Stock Center. We also constructed *35S*:*ZFP3* transgenic lines and selected two representative lines, which showed higher level of *ZFP3* expression in comparison to the wild type (Fig 1). Seeds of *zfp3*, *35S*:*ZFP3-6*, *35S*:*ZFP3-11* and wild type were geminated on 1/2 MS medium with 0, 100, 150 and 200 mM NaCl. No significant difference among wild type, mutant and overexpression line was observed when seeds were grown on 1/2 MS medium (Fig 2a). When treated with 100 mM NaCl, *zfp3* mutant exhibited significantly lower germination rates than wild type, while *ZFP3* overexpression lines displayed significantly higher germination rates. However, as the concentration increases, the germination rates were decreased dramatically. The decrease in germination rates was more significant upon exposure to 150 and 200 mM NaCl. At the 3rd day and 4th day post germination on 150 mM NaCl, the rates of germination in *zfp3* were significantly delayed compared to wild type. But *35S*:*ZFP3-6* and *35S*:*ZFP3-11* lines germinated significantly earlier than wild type (Fig 2c). On 200 mM NaCl medium, the differences of germination rate of wild type, *zfp3*, *35S*:*ZFP3-6* and *35S*:*ZFP3-11* were significant from the 3rd to7th day post germination (Fig 2d). To comprehend the role of *ZFP3* in osmotic stress, we also examined the germination condition on 1/2 MS medium supplemented with different concentrations of mannitol and sucrose. As shown in Fig 3a to 3d, germination rates among the *zfp3* mutant, *ZFP3* overexpression lines and wild type were not significantly different on 1/2 MS medium. However, the *zfp3* mutant displayed a significant decrease in germination rate compared to the wild type on 300, 400 and 500 mM mannitol. Contrary to *zfp3* mutant, the overexpression lines showed significantly increased germination rates in these treatments. A similar trend in germination rates was observed on mediums containing different concentrations of sucrose. The germination rates were significantly lower in *zfp3* mutant and significantly higher in *ZFP3* overexpression lines in comparison to wild type when treated with 200 mM sucrose. Upon exposure to 250 and 300 mM sucrose, the *zfp3* mutant exhibited significant decrease in germination rates during most of exposure period (Fig 4a–4d). Meanwhile, when treated with 300 mM sucrose, the overexpression lines showed significantly higher germination rates than wild type from the 2nd to 7th day post germination (Fig 4d). The results indicate that loss of function of *ZFP3* reduces the tolerance to salt and osmotic stress while overexpression of *ZFP3* exhibited an increased tolerance to salt and osmotic stress in *Arabidopsis thaliana*.

To further confirm this conclusion, root length of the mutant, overexpression line and wild type were measured on different treatments. No significant difference in root length was recorded among wild type, *zfp3*, *35S*:*ZFP3-6* and *35S*:*ZFP3-11* seedlings on 1/2 MS mediums (Figs 2e and 2f, 3e and 3f, 4e and 4f). When treated with 100 and 150 mM NaCl, significant inhibition in root lengths was observed. The average root length of *zfp3* seedlings was significantly shorter than wild type seedlings on 100 mM and 150 mM NaCl. Nevertheless, the average root lengths of *35S*:*ZFP3* seedlings were significantly longer in comparison to wild type seedlings (Fig 2e and 2f). When exposed to mannitol and sucrose, the *zfp3* mutant was also more sensitive than wild type on root length. When treated with 200 mM mannitol, the root length of *zfp3* seedlings was decreased significantly in comparison to wild type. However, *35S*:*ZFP3-6* and *35S*:*ZFP3-11* lines were about 16% longer than wild type (Fig 3e and 3f). On 300 mM mannitol medium, the root length of *zfp3* seedlings was 16% shorter than wild type seedlings and that of *ZFP3* overexpression lines seedlings were significantly longer than wild type seedlings (Fig 3e and 3f). The data displayed that root length of *zfp3* mutant was significantly shorter than wild type under different sucrose treatments. Root length of *zfp3* seedlings was shorter in comparison to wild type on both 150 mM and 250 mM sucrose medium (Fig 4e and 4f). Oppositely, root length of *35S*:*ZFP3-6* line was 16.9% and 17.5% longer than that of wild type (Fig 4e and 4f). Taken together, these data suggest that *ZFP3* is a key regulator controlling salt and osmotic stress response in *Arabidopsis*.

### *ZFP3* expression is suppressed by salt and osmotic stress

**Fig 5:**
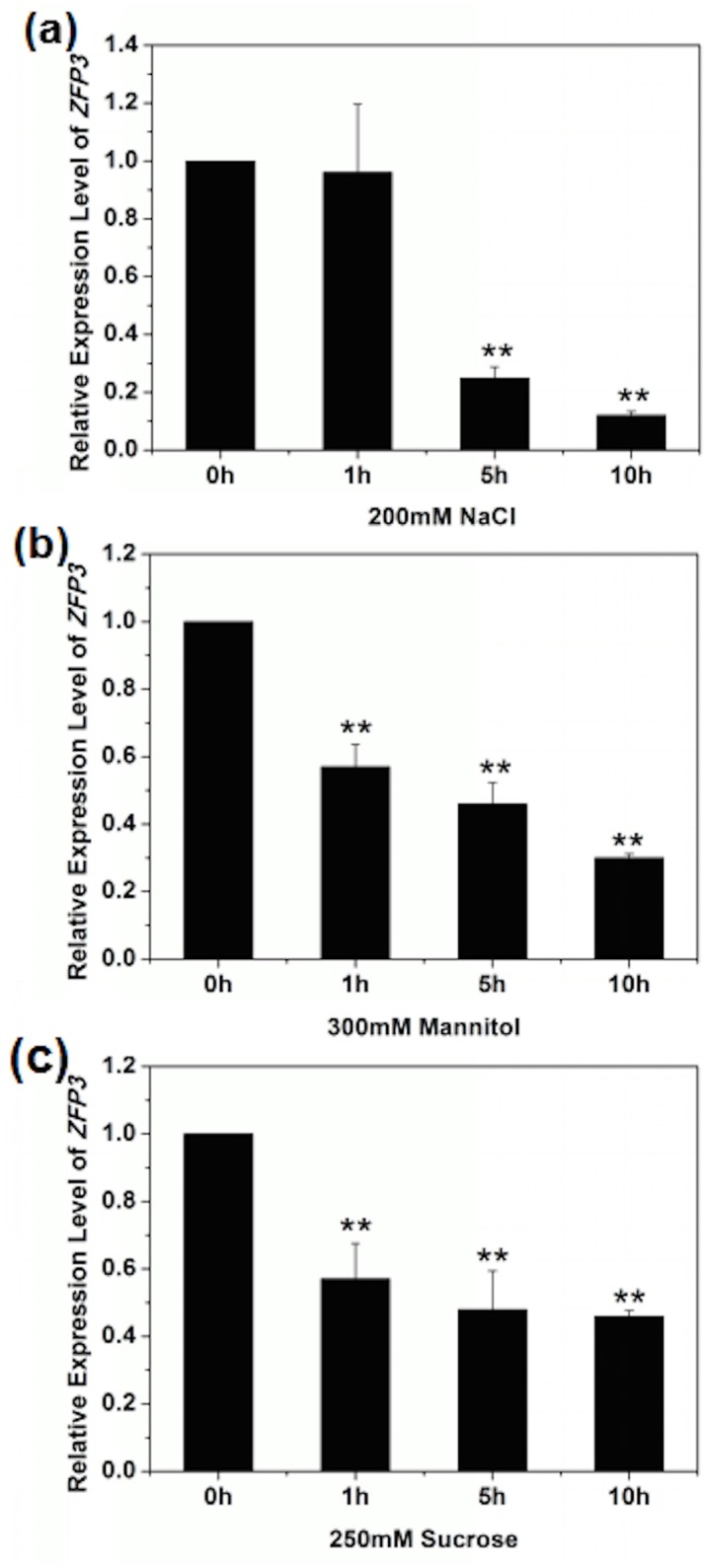
*ZFP3* Expression was suppressed by salt and osmatic stress. **(a)** Expression level of *ZFP3* treated with 200 mM NaCl at different time points. **(b)** Expression level of *ZFP3* treated with 300 mM mannitol at different time points. **(c)** Expression level of *ZFP3* treated with 250 mM sucrose at different time points. The values were normalized against the levels of *TUB2* as a control. Error bars indicate SD. *, *P*<0.05; and **, *P*<0.01(Student’s t-test).

In order to further confirm the function of *ZFP3* in abiotic stress, real-time PCR analyses were done to detect the expression of *ZFP3* gene under different concentrations of NaCl, mannitol and sucrose in wild type seedlings. As shown in Fig 5a, the expression level of *ZFP3* was dramatically reduced to 0.12 fold after 10 hours of NaCl treatment. Similarly, the expression levels of *ZFP3* were also rapidly induced by mannitol (Fig 5b) and sucrose (Fig 5c), which displayed a gradual decrease and only reached 0.30 and 0.46 of initial level after 10 hours. Taken together, these results confirm that *ZFP3* expression is suppressed by abiotic stress response.

### *ZFP3* affects proline and chlorophyll contents under salt stress and osmotic stress

**Fig 6:**
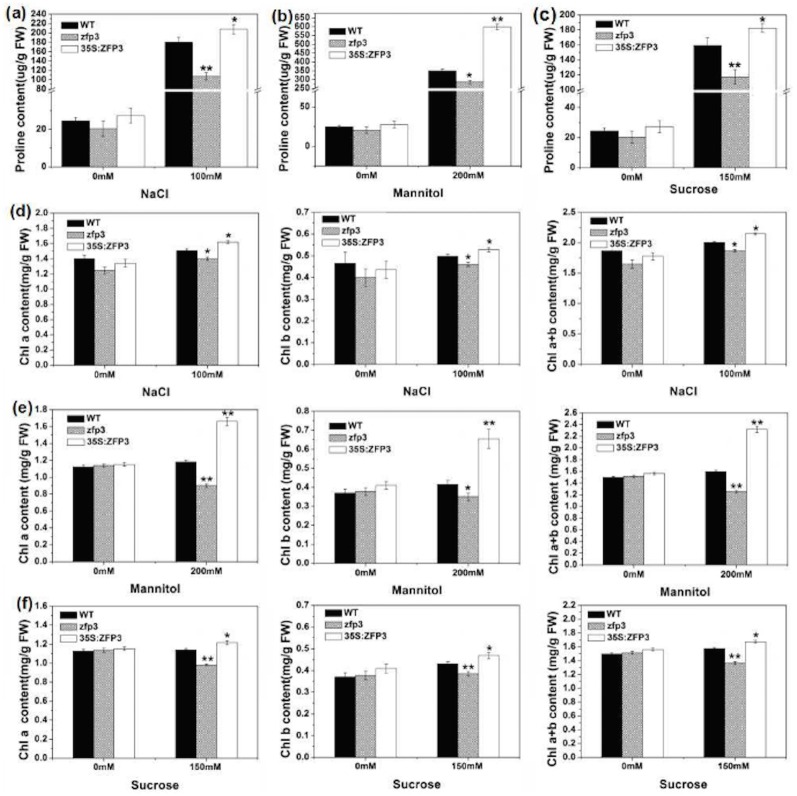
Analyses of proline and chlorophyll content in WT, *zfp3* and *35S*:*ZFP3* under salt and osmotic stress. **(a to c)** Proline content of three materials without or with 100 mM NaCl **(a)**, 200 mM mannitol **(b)** and 150 mM sucrose **(c)**. **(d to f)** Chlorophyll a, chlorophyll b and chlorophyll a+b content of three materials without or with 100 mM NaCl **(d)**, 200 mM mannitol **(e)** and 150 mM sucrose **(f)**. Error bars indicate SD. *, *P*<0.05; and **, *P*<0.01(Student’s t-test).

Proline, which works as a kind of compatible osmolyte, plays an important role in protecting plants from abrasive environmental stresses especially osmotic and salt stresses [36–40]. The content of proline was determined in wild type, *zfp3* mutant and *ZFP3* overexpression line (*35S*:*ZFP3-6*) with 0 mM, 100 mM NaCl, 200 mM mannitol and 150 mM sucrose treatments. As shown in Fig 6a–6c, the contents of proline were not significantly different among these three lines under normal environmental conditions. However, the overexpression line accumulated more proline than wild type, while the proline content of *zfp3* mutant was significantly less than wild type when treated with NaCl, mannitol and sucrose (Fig 6a–6c).

Then, we further determined the chlorophyll contents of wild type, *zfp3* and *35S*:*ZFP3 Arabidopsis* plants under different conditions. Under normal condition, there were no significant differences among wild type, *zfp3* and *35S*:*ZFP3* (Fig 6d and 6e). Chlorophyll a, chlorophyll b and chlorophyll a+b contents of *zfp3* were decreased significantly compared to wild type under high concentration of NaCl, mannitol and sucrose conditions. However, higher chlorophyll content was found in overexpression line in comparison to wild type (Fig 6d and 6e).

### *ZFP3* regulates the expressions of stress-related genes

**Fig 7:**
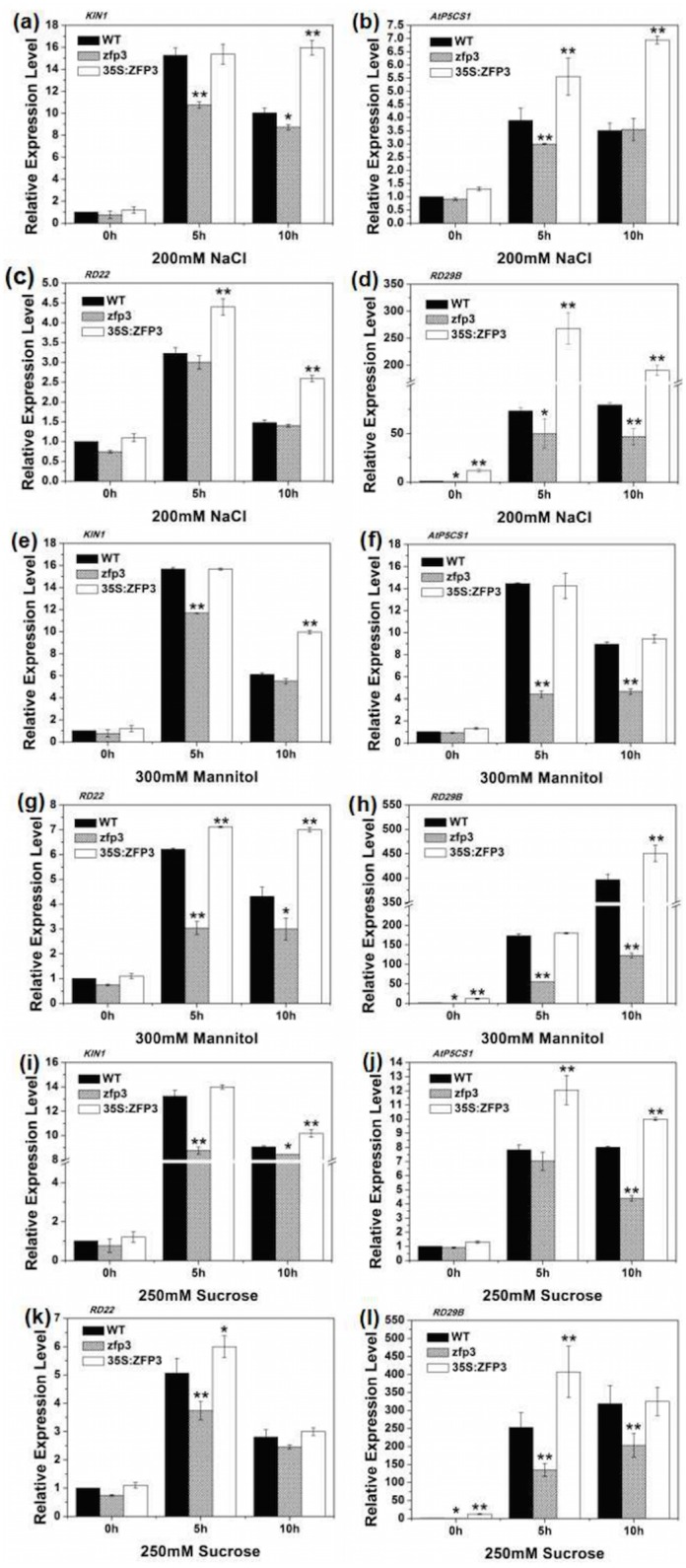
Expression levels of stress-related genes in *zfp3* mutant and overexpression line. **(a to d)** Quantitative RT-PCR analysis of expression level of four stress-related genes, *KIN1* **(a)**, *AtP5CS1* **(b)**, *RD22* **(c)** and *RD29B* **(d)**, in WT, *zfp3* mutant and *35S*:*ZFP3* seedlings treated with or without 200 mM NaCl. **(e to h)** Quantitative RT-PCR analysis of expression level of four stress-related genes, *KIN1* **(e)**, *AtP5CS1* **(f)**, *RD22* **(g)** and *RD29B* **(h)**, in WT, *zfp3* mutant and *35S*:*ZFP3* seedlings treated with or without 300 mM mannitol. **(i to l)** Quantitative RT-PCR analysis of expression level of four stress-related genes, *KIN1* **(i)**, *AtP5CS1* **(j)**, *RD22* **(k)** and *RD29B* **(l)**, in WT, *zfp3* mutant and *35S*:*ZFP3* seedlings treated with or without 250 mM sucrose. The values were normalized against the levels of *TUB2* as a control. Error bars indicate SD. *, *P*<0.05; and **, *P*<0.01(Student’s t-test).

To further investigate the functions of *ZFP3* in abiotic stresses, we analyzed the expressions of some stress-related marker genes such as *KIN1* (*At5g15960*), *RD22* (*At5g25610*), *RD29B* (*At5g52300*) and osmotic substance synthesis gene *AtP5CS1* (*At2g39800*) that encodes pyrroline-5-carboxylate synthetase [15,38]. The plants of wild type, *zfp3* mutant and *ZFP3* overexpression line (*35S*:*ZFP3-6*) were treated with 200 mM NaCl, 300 mM mannitol and 250 mM sucrose for 5h and 10h respectively. As shown in Fig 7, for the normal 1/2 MS medium, there were no significant differences in all the genotypes in the expression pattern of four stress-related genes except *RD29B*. However, the expressions of these genes in wild type, *zfp3* mutant and overexpression line were all significantly induced when treated by NaCl, mannitol and sucrose. The expressions of these genes in overexpression line were significantly higher than wild type while the expressions of these genes in *zfp3* mutant were significantly lower than wild type (Fig 7). These results suggest that *ZFP3* acts upstream of these genes and further research on these genes interact with *ZFP3* to regulate network in salt stress and osmotic stress response is needed.

## Discussion

In *Arabidopsis* C_2_H_2_-type ZFPs consists of 176 members and is one of largest transcription factor families. The cDNA of *ZFP3* is 959 bp and encodes a zinc finger protein containing only a single zinc finger. C_2_H_2_-type ZFPs play a major role in growth and development of plants [30]. It was reported that they participate in various biological processes including signal transduction, DNA/RNA binding, morphogenesis, transcription regulation, abiotic and biotic response [4,16–19,41]. Our previous work also have demonstrated that C2H2-type ZFPs GIS, GIS2, GIS3, ZFP8, ZFP5 and ZFP6 are all C_2_H_2_-type ZFPs control the trichome initiation by integrating GA and Cytokinin signals [18–21,23,42,43]. *AtZFP1* was reported to plays a role in downstream of photomorphogenic activation [44]. Overexpression of *ZFP2* was shown to delay flower abscission and *ZFP2* may influence organ shed directly or indirectly in *Arabidopsis* [45]. An important feature of *ZFP3* is the presence of a zinc finger motif. This motif is very conservative in both monocots and dicots, which indicate that they may have the similar biological function [46]. It was shown that ZFP3 protein is localized in nucleus, which suggests its potential functional diversity. *ZFP3* was previously shown to regulate light and ABA signaling in seeds germination and plant development in *Arabidopsis* [31]. To further examine the function of *ZFP3*, we studied the phenotype of wild type, *zfp3* mutant and *ZFP3* overexpression line under stress condition. Our results demonstrated that *ZFP3* is a key regulator controlling plants in response to salt stress and osmotic stress (Figs 2–4).

Chlorophyll content is an important parameter to determine the capacity of plant photosynthesis. Analysis of chlorophyll content revealed that the content of *ZFP3* overexpression line is significantly higher compare to wild type while the mutant displayed an opposite result under stress conditions. Proline accumulation is a widespread phenomenon when plants suffer from a variety of hostile environmental stress [47]. Furthermore, proline is also known as a compatible osmolyte which can scavenge free radicals, keep the balance of intracellular redox, stabilize subcellular structures during stress, especially salt and osmotic stress [38,39]. In this study, the proline accumulation is elevated significantly in all of three lines. However, the elevated level of *zfp3* mutant is much lower than that of wild type, while overexpression line is relatively higher in salt and osmotic stress (Fig 6a–6c). Proline is synthesized from glutamate or ornithine. In glutamate pathway, there are two steps in which glutamate is converted to glutamyl-5-semialdehyde (G5SA) by Δ^1^- pyrroline-5-carboxylate synthase (P5CS) and G5SA is converted to proline by the Δ^1^-pyrroline-carboxylate reductase (P5CR). Among them, the γ-glutamyl kinase activity of P5CS is the key rate-limiting factor [36–38,48,49]. Therefore we investigated the expression level of *AtP5CS1*. Our results show that the expression level of *AtP5CS1* was significantly higher in overexpression line while the expression of this gene was significantly lower in the *zfp3* mutant in comparison to wild type (Fig 7b, 7f and 7j).

To further test the change of expression level in stress pathway genes, we analyzed several stress marker genes including *KIN1*, *RD22* and *RD29B* (Fig 7). The results also demonstrated *ZFP3* affects plants’ tolerance to salt and osmotic stress by regulating the expressions of these genes. However, the mechanism of *ZFP3* interacts with these genes controls salt and osmotic stress should be further investigated.
